# Diabetes‐induced vascular calcification is associated with low pyrophosphate and its oral supplementation prevents calcification in diabetic mice

**DOI:** 10.1002/2211-5463.70141

**Published:** 2025-10-13

**Authors:** Krisztina Fülöp, Eszter Kozák, Natália Tőkési, Lilian Kocsis, Anikó Kelemen, Zsuzsanna Geszti, Adriána Kutás, Mariann Harangi, Ágnes Diószegi, Zsolt Rapi, József Balla, Olivier Le Saux, András Váradi, Viola Pomozi

**Affiliations:** ^1^ Institute of Molecular Life Sciences, Research Centre for Natural Sciences, Centre of Excellence of the Hungarian Academy of Sciences, HUN‐REN Budapest Hungary; ^2^ Division of Metabolism, Department of Internal Medicine, Faculty of Medicine University of Debrecen Debrecen Hungary; ^3^ Vascular Pathophysiology Research Group 11003 University of Debrecen, HUN‐REN Debrecen Hungary; ^4^ Department of Organic Chemistry and Technology Budapest University of Technology and Economics Budapest Hungary; ^5^ Division of Nephrology, Department of Internal Medicine, Faculty of Medicine University of Debrecen Debrecen Hungary; ^6^ Department of Cell and Molecular Biology, John A. Burns School of Medicine University of Hawaii Honolulu HI USA

**Keywords:** cardiovascular calcification, diabetes, mouse model, pyrophosphate

## Abstract

The predominant cause of death among diabetic patients comes from cardiovascular complications, including vascular calcification. The objectives of this study were to improve the understanding of the molecular mechanisms involved in diabetes‐related calcification and to test potential preventive therapies. We found that levels of plasma pyrophosphate—a potent inhibitor of calcification—were decreased in type 1 and type 2 diabetic patients with cardiovascular symptoms. To further investigate vascular calcification, we developed a diabetic mouse model that showed increased aorta and renal calcification compared to control. Alkaline phosphatase activity was also increased in the circulation of diabetic mice, which resulted in a significant decrease in plasma pyrophosphate. Oral treatment with pyrophosphate prevented diabetes‐induced calcification in mice, providing a direct translational value for clinical applications.

## Graphical abstract

Induction of diabetes in three different mouse strains uniformly resulted in an increase in TNAP activity and a reduction in pyrophosphate (PPi) in the circulation. Inhibition of TNAP restored plasma PPi. Diabetes‐induced calcification in the media layer of the aorta was detected only in the Abcc6−/− strain, which is predisposed to ectopic calcification. Orally given PPi rescued the calcification phenotype.

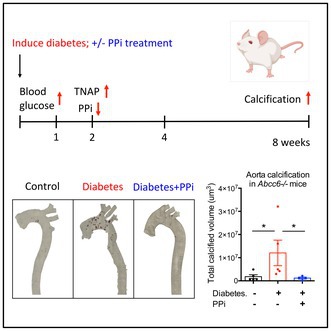

**Abbreviations**
ABCC6: ATP Binding Cassette Subfamily C Member 6
ALP: alkaline phosphatase
ATP: adenosine triphosphate
DKD: diabetic kidney disease
DM: diabetes mellitus
ENPP1: ectonucleotide pyrophosphatase/phosphodiesterase 1
GACI: generalized arterial calcification of infancy
PPi: inorganic pyrophosphate
PXE: pseudoxanthoma elasticum
STZ: streptozotocin
T1D: type 1 diabetes
T2D: type 2 diabetes
TNAP: tissue nonspecific alkaline phosphatase

About 10% of the population has diabetes mellitus (DM), and the prevalence of diabetes keeps rising (according to the International Diabetes Federation). Type 1 (juvenile onset) diabetes (T1D) is caused by an autoimmune response against insulin‐producing beta cells [1], while in Type 2 (adult onset) diabetes (T2D), insulin deficiency and/or resistance progressively develop [2]. Cardiovascular manifestations are the leading cause of death among adults with DM and diabetic kidney disease (DKD), and arterial calcification is a major contributing factor [3, 4]. Ectopic calcification was long thought to result from passive formation of hydroxyapatite crystals. However, it has now become clear that calcification results from loss of inhibition and/or enhanced induction of osteogenic differentiation [5].

Vascular calcification can be distinguished as intimal or medial calcification. The latter is defined as calcification within the medial layer of the arterial wall between smooth muscle cells [6]. Medial arterial calcification is often associated not only with diabetes but also with aging and renal failure [7]. Intimal calcification develops in the inner arterial layer and is usually associated with atherosclerotic plaques [8]. Intimal and medial calcification can be present separately or together in DM; however, medial calcification is more prevalent [4, 9]. Diabetes‐related calcification affects different arterial beds, mainly the aorta [10], the coronary arteries [11] as well as peripheral [12] and renal arteries [13].

In DM, a chronic state of hyperglycemia and other metabolic disturbances leads to the activation of various pathways that ultimately promote calcification. These pathways include dysregulated mineral and lipid metabolism, inflammation, oxidative stress, and impaired vascular smooth muscle cell (VSMC) function [14, 15, 16].

The role of bone‐specific and intestinal alkaline phosphatase (ALP) in diabetic patients has been investigated in a few studies [17, 18]. Serum ALP level was proposed as a predictor of long‐term risk of all‐cause and cardiovascular morbidity in DM [19]. Importantly, ALP hydrolyzes pyrophosphate (PPi), which is a prevalent and potent inhibitor of soft tissue calcification [20]. In recent years, the identification of mutations in *ABCC6* [21, 22, 23] and the characterization of its function [24] have provided novel insights into the regulation of ectopic calcification in relation to PPi. ABCC6 mediates the cellular efflux of ATP, which is sequentially converted into PPi and adenosine monophosphate and then to adenosine at the cellular surface by the ectonucleotidases NPP1 (encoded by *ENPP1*) and CD73 (encoded by *NT5E*), respectively [24, 25]. PPi and indirectly adenosine are key molecules in the prevention of mineralization in soft tissues [20, 26]. Reduced or absent PPi production causes the calcification disorders pseudoxanthoma elasticum (PXE, MIM#264800, *ABCC6* mutations) and generalized arterial calcification of infancy (GACI, MIM#208000, *ENPP1* mutations) [24, 25, 27, 28]. Adenosine (produced through enzymatic cleavage of AMP by CD73) is an important inhibitor of TNAP activity. Decreased adenosine levels caused by *NT5E* (CD73) mutations underlie the calcification of joints and arteries (CALJA, MIM#211800) where the observed ectopic mineralization occurs due to enhanced PPi degradation from increased ALP activity [29]. Furthermore, decreased plasma PPi levels were found in other disorders that manifest with ectopic calcification, such as systemic sclerosis [30], in patients with kidney failure undergoing dialysis [31, 32] and in a mouse model of progeria [33]. ABCC6 appears to play a role in vascular calcification in chronic kidney disease in both animal models and humans [34, 35] and a negative correlation between serum ALP activity and plasma PPi levels was also observed [31]. Inhibiting ALP attenuated vascular calcification in animal models of chronic kidney disease‐mineral and bone disorder [36] and PXE [37]. In a recent study, a direct association was found between elevated blood glucose level and changes in PPi metabolism [38]. These findings highlight the importance of PPi in regulating ectopic calcification in a variety of pathologies; however, its precise role in diabetes‐associated calcification is still poorly understood.

In this study, our aims were to (i) test our hypothesis that plasma PPi level is lower in diabetic patients; (ii) develop a novel mouse model showing diabetes‐related soft tissue calcification; (iii) investigate the molecular background of diabetes‐related calcification, with a main focus on the PPi pathway; and (iv) test the efficacy of PPi supplementation in the prevention of diabetes‐related calcification.

## Materials and methods

### Research design

Human blood samples were collected with informed written consent of the participants. For the mouse model, diabetes was induced by streptozotocin (STZ) injection in wild‐type mice and a mouse strain susceptible to ectopic calcification. Mice were terminated at different time points, and several tissue samples were collected to assess soft tissue calcification and measure calcification‐related parameters. For human plasma PPi measurements, blood was collected from male T1D and T2D patients and from healthy male volunteers. For the *in vivo* experiments, *Abcc6−/−*, C57BL/6 (*Abcc6+/+*), and CD1 mice were used (described in detail below in the “Human cohort” and “Animals” sections). Human samples were age‐matched with controls. Sample sizes were selected based on previous experience with similar methods. Details on sample sizes representing biological replicates are shown in the figure legends. For plasma PPi, plasma ALP activity, and RT‐PCR measurements, samples were measured in triplicates. For calcium content determination by colorimetric assay, samples were measured twice. Mice showing nondiabetes‐related pathological symptoms (e.g., spontaneous tumor growth or chronic inflammation) were terminated and not used in the experiments, but otherwise, no samples or data were excluded from the study. Mice were grouped such that the average weight would be similar across the different conditions. Conditions were assigned randomly to groups. Animal studies were not blinded; data analysis was blinded.

### Human cohort

Permission to collect blood samples from diabetic patients and healthy volunteers was granted by the Regional Ethics Committee of the University of Debrecen and the Medical Research Council (registration numbers: DE RKEB/IKEB 5513B‐2020 and IV/7989–1/2020/EKU, respectively). Informed written consent was obtained from each participant prior to the study, and experiments conformed to the principles of the Declaration of Helsinki, as indicated in the above document. All patient samples were handled in an anonymized form, which was also approved by the above document.

Recruitment of diabetic patients and age‐ and sex‐matched healthy controls was coordinated by the University of Debrecen, Hungary. Both T1D and T2D patients were included in the study. PPi has a crucial role in bone metabolism, and estrogen and menopause have significant effects on bone mineral density and bone structure, which might affect plasma PPi levels. To avoid this possible confounding factor, only male patients and healthy controls were recruited in this study; however, we expect similar changes in females as well.

Blood samples were taken for PPi assay in the morning between 7 and 9 am after overnight fasting from diabetic patients and from nondiabetic volunteers.

### Animals

The animal studies were approved by the RCNS Institutional Animal Care and Use Committees (Permit number: PE/EA/748–2/2021) and were conducted according to national guidelines.

*Abcc6* ^*tm1Aabb*^ mice were generated on a 129/Ola background [39] and backcrossed into a C57BL/6J more than 10 times. These mice are herein designated *Abcc6−/−*. The *Abcc6−/−* mice were obtained from Dr. Olivier Le Saux, University of Hawaii via a conventional MTA. *Abcc6−/−* mice lack the Abcc6 protein and develop phenotypic calcification similar to human PXE patients affecting arteries, eyes, renal, as well as the capsule of the vibrissae in the whiskers [39, 40, 41].

C57BL/6J mice designated as wild‐type were derived from mice purchased from The Jackson Laboratories. CD1 mice were a generous gift from Prof. Frank Rutsch. Both male and female, age‐matched mice were used. All animals were housed in approved animal facilities at the HUN‐REN Research Centre for Natural Sciences. At the start of the experiments, all mice were healthy and in the normal weight range. Mice were kept under routine laboratory conditions with a 12‐h light–dark cycle with *ad libitum* access to water and chow. At the end of the experiment, the mice were sacrificed with an overdose of anesthetics: Zoletil (60 mg/kg), Xilazin (25 mg·kg^−1^), and Butorfanol (6 mg·kg^−1^).

### Diabetic mouse model: STZ treatment

A well‐known agent to chemically induce type 1 diabetes by beta cell depletion is streptozotocin (STZ) [42]. Based on [43] with some modifications, CD1 mice received a single dose of 75 mg·kg^−1^, and *Abcc6+/+* and *Abcc6−/−* mice received 150 mg·kg^−1^ intraperitoneal injection of streptozotocin (Tocris, 1621; Bristol, UK), dissolved in 10 mm citric acid. If mice did not reach diabetic blood glucose level 5 days after STZ injection, a second IP injection of the same dose was administered.

Mice were kept in diabetic condition for 2, 4, 8, 16, or 20 weeks. In the case of *Abcc6−/−* and *Abcc6+/+* mice, STZ treatment was applied at the age of 15 weeks. STZ treatment of CD1 mice was timed so that all groups were 12 months old by the end of the experiment.

### PPi treatment

Based on our earlier work [44], mice in the PPi‐treated group were given 0.3 mm Na_2_H_2_P_2_O_7_ (Sigma Aldrich, 71 501; Saint Louis, MO, USA) in drinking water from the time of STZ injection throughout the experiment.

### ALP inhibitor treatment

18‐week‐old *Abcc6−/−* mice were kept in diabetic condition for 2 weeks and treated with 5 mg·kg^−1^·day^−1^ SBI‐425 TNAP inhibitor in the last 2 days of the experiment. (SBI‐425 was synthesized by Zsolt Rapi at the Budapest University of Technology and Economics in collaboration with BrainVisionCenter Ltd.). SBI‐425 was administered via oral gavage after 2 h of fasting. Mice were sacrificed 90 min after the SBI‐425 administration, and heparin/CTAD anticoagulated plasma samples were collected for ALP/PPi measurements, respectively.

### Insulin replacement

To test the effect of insulin on plasma ALP activity, animals reaching a 16 nmol·L^−1^ blood glucose level received 4 μU·g^−1^ long‐acting basal insulin (Sanofi, Insuman Basal SoloStar: Paris, France) IP injection twice a day for 3 weeks. After the mice were sacrificed, heparin anticoagulated plasma samples were collected for ALP measurements.

### Detection of calcification

#### Aorta calcification

Aorta preparation: The ascending and descending aorta (from the heart to the diaphragm) was dissected from the sacrificed mice and fixed in 96% ethanol for 48 h at room temperature.

Aorta staining: Aortas were stained with 0.003% Alizarin red S (Sigma Aldrich, A5533) dissolved in 1% KOH for 48 h at room temperature.

Aorta imaging: Before imaging, samples were rinsed in 1% KOH for 5 min and then placed on microscopic slides, mounted in 1% KOH and closed with coverslip. Images were taken using a Leica SP8 confocal microscope using a HC FLUOTAR 10× 0.32DRY objective. To detect Alizarin red signal the 488 nm laser was used for excitation and the emission was detected between 603 and 778 nm. For background correction the autofluorescence of the samples was detected between 492 and 573 nm. For the quantification of the calcification XYZ direction tile scan was performed on the whole aortas, with equal overlap between slices. Slice resolution: 512 × 512 pixels, 8 bit.

3D image analysis: Data analysis was performed by ImageJ 1.53t version. The image series (108 image·sample^−1^) was imported as LIF file format (Leica Image File) where the spatial calibrations are automatically imported, and the full metadata is displayed. After removing background noise (gaussian filter, sigma = 1) of the image, the potential contaminants were removed using the “Subtract create stack” ImageJ function. The quantification was based on connectivity analysis, and an open‐source “3D Object Counter” plugin (min object size = 100 μm, threshold = 5) was used to determine the calcified parts in the aorta. The internal macro programming language of ImageJ was used to automate the preprocessing and analysis steps of the microscope images. During preprocessing of the image, an intensity histogram graph was used for validation to avoid over‐processing of the image. For quantification validation, the results generated by the 3D Object Counter plugin were compared with manual evaluation. Both diabetic and control samples were used to run the automated script and evaluate the results.

Cross section: aorta stained with Alizarin Red was embedded in Shandon Cryomatrix (Thermo Scientific, 6 769 006); then, 20‐μm‐thick sections were cut using a cryomicrotome. Sections were analyzed and images were taken using a Leica DMLS microscope equipped with C PLAN 10x/0.1 and 40×/0.1 objectives. Images were acquired by a CCD camera (Imaging Source, Nikon) controlled by the Windows version of NIS (version 5.21.02, Nikon).

#### Kidney and vibrissae capsule calcification

To quantify the levels of mineral deposition in the kidneys and in the whiskers, we carried out a colorimetric assay [45] that measures directly the amount of calcium within excised tissue. Briefly, one kidney or one whisker was harvested, minced, and incubated at room temperature for 48 h in 200 μL 0.15 N HCl. The total calcium content of the HCl supernatant was assessed by measuring the absorbance at 550 nm using the Stanbio Calcium LiquiColor kit (Thermo Fisher Scientific, SB‐0150‐250; Waltham, MA, USA). The obtained absorbance values were quantified against a known standard to provide calcium concentration in mg per ml.

### Plasma PPi, urea and creatinine levels

For measuring PPi level in plasma, CTAD blood was collected from mice via cardiac puncture and from humans via venous blood sampling. Plasma was separated by centrifugation and transferred into separation tubes (Sartorius Centrisart 1, 300,000 MW, 13279E; Göttingen, Germany) and filter‐centrifuged. PPi was first converted to ATP by adenosine 5′‐triphosphate sulfurylase (ATPS) (ProSpec, ENZ‐353; Rehovot, Israel) in the presence of excess adenosine 5′‐phosphosulfate (APS) (Santa Cruz Biotechnology, sc‐214 506; Dallas, TX, USA). The concentration of ATP generated from PPi was measured using the BacTiter‐Glo kit (Promega, G8230; Madison, WI, USA) [25].

The levels of carbamide and creatinine in the plasma, indicators of renal function, were determined by the Department of Clinical Pathology and Oncology of the University of Veterinary Medicine Budapest.

### Urine volume

In order to collect urine samples, 2–4 mice were housed in metabolic cages for 24 h. The volume of urine was measured after 24 h and was divided by the number of animals in the cage.

### Plasma ALP activity

Heparin treated blood samples were centrifuged at 1000 g for 10 min at 4°C, and the supernatant was stored at −80°C. ALP activity was measured in triplicates. Samples were diluted 10 times in the assay buffer, and 10 μL in 150 μL final assay volume was measured using 4‐methylumbelliferyl phosphate disodium salt (MUP) as substrate with Abcam 83 371 Kit on PerkinElmer IsoplateTM‐96F black frame plates (PerkinElmer, Waltham, MA, USA). Fluorescence intensity was detected in Enspire Multimode Plate Reader (PerkinElmer, Waltham, MA, USA) (Ex/Em = 360 nm/440 nm) after 30‐min incubation at 25°C. For the SBI‐425 inhibitor assay, heparin plasma samples were diluted three times in the assay buffer.

### RT‐PCR

We used quantitative real‐time PCR to determine the level of mRNA expression of *Abcc6* and *Enpp1* in the liver. *Gapdh* was used as a housekeeping control. (TaqMan probes: Thermo Fisher Scientific, Gapdh: Mm99999915_g1, Abcc6: Mm00497698_m1, Enpp1: Mm01193761_m1; Waltham, MA, USA).

Total RNA was extracted from tissue samples using Trizol reagent (Thermo Fisher Scientific, 15 596 026; Waltham, MA, USA) [46]. RNA was converted into first‐strand cDNA using SuperScript™ III First‐Strand Synthesis System with random hexamers (Thermo Fisher Scientific, 18 080 051; Waltham, MA, USA). Gene expression levels were detected by quantitative RT‐PCR with a StepOnePlus Real‐Time System (Applied Biosystems, Waltham, MA, USA).

### Statistical analysis

Data analysis and visualization were performed using Prism 6.0 (GraphPad Software). Data were analyzed by two‐tailed Mann–Whitney nonparametric tests, except for RT‐PCR results. Values are expressed as mean ± standard error of the mean (SEM). A *P* < 0.05 was considered statistically significant. *P* values are denoted with asterisks: *P* > 0.05, ns; *P < 0.05; **P < 0.01; ***P < 0.001; and ****P < 0.0001.

Relative gene expression from the RT‐PCR results was calculated using the delta–delta threshold method. Data were analyzed by Student's t‐test. Differences between relative gene expressions > 1.5‐fold were considered statistically significant.

Animal numbers used for individual datasets varied and are shown in the figure legends.

## Results

### Plasma PPi is decreased in human diabetic patients and in diabetic mice

Since lower plasma PPi is associated with several diseases with ectopic calcification symptoms and elevated blood glucose levels were shown to disrupt extracellular PPi metabolism [38], our central hypothesis was that DM‐associated arterial calcification could be related to a deficit in pyrophosphate. Therefore, we measured plasma PPi levels of T1D and T2D patients and healthy controls. Sixty‐one diabetic patients were recruited, 12 with type 1 and 49 with type 2 diabetes. We selected patients with cardiovascular complications, and the average age was 70. The cardiovascular symptoms included 38 cases of coronary artery disease, 19 cases of acute myocardial infarction, 8 cases of ischemic stroke, 22 cases of carotid artery stenosis, 21 patients with peripheral arterial disease, and 57 patients with hypertension—see Table S1.

**Fig. 1:**
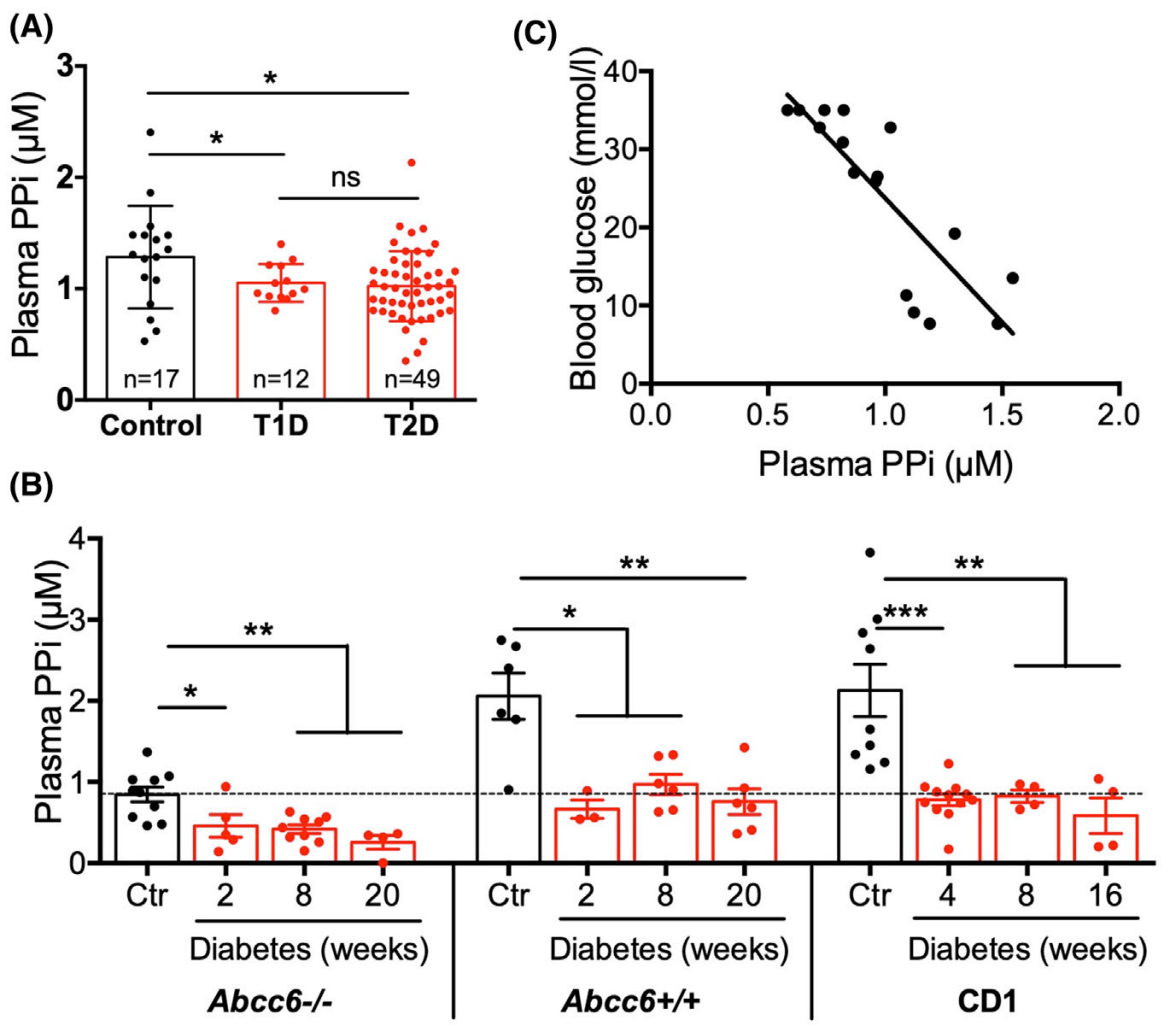
Plasma PPi levels in humans and mice. (A) Plasma PPi levels are decreased in T1D and T2D patients compared to healthy controls. Data were analyzed by two‐tailed Mann–Whitney nonparametric tests, and results are expressed as mean ± SEM; ns, not significant, *P < 0.05 (*n* = 17 Control, 12 T1D, 49 T2D). (B) PPi level decreased in the plasma of *Abcc6−/−, Abcc6+/+*, and in CD1 mice 2 weeks after the onset of diabetic condition compared to their nondiabetic controls. Data were analyzed by two‐tailed Mann–Whitney nonparametric tests, and results are expressed as mean ± SEM; *P < 0.05; **P < 0.01; ***P < 0.001 (*n* = 3–10). (C) Analysis of blood samples from age‐matched diabetic and nondiabetic *Abcc6+/+* mice showed inverse correlation between blood glucose and plasma PPi levels (*n* = 16), Pearson correlation coefficient = −0.8356, *P* < 0.0001 (PPi, pyrophosphate).

We found that plasma PPi level in healthy controls was 1.29 ± 0.11 μmol·L^−1^, while in T1D and T2D patients it was 1.05 ± 0.05 μmol·L^−1^ and 1.02 ± 0.04 μmol·L^−1^, respectively. Therefore, both T1D and T2D patients have ~25% less plasma PPi compared to age‐matched healthy controls (Fig. 1A). Plasma ALP activity in T1D and T2D patients (96.14 ± 15.47 and 78.77 ± 5.23 U·L^−1^, respectively) was within the normal range of 40–115 U·L^−1^ (Table S1).

To investigate DM‐associated calcification and the potential role of PPi in diabetes, we generated a type 1 diabetic mouse model. We have been investigating mice with three different genetic backgrounds: (i) *Abcc6−/−* mice, susceptible to late calcification; (ii) *Abcc6+/+* (C57BL/6) mice as a control for *Abcc6−/−*; (iii) CD1 strain, an additional unrelated wild‐type control.

Diabetes was induced by streptozotocin injection, which resulted in successful destruction of beta cells and led to hyperglycemia in all three mouse models. Blood glucose level rose above 16 mmol·L^−1^ within 2 weeks of injection (Fig. S1A). Increased urination is a typical symptom of diabetes, and the STZ‐induced diabetic mice also showed polyuria (Fig. S1B). As diabetes often leads to kidney disease (DKD), we also measured plasma urea and creatinine levels. Elevation of these values reflects a reduction in the filtration capacity of the glomeruli and indicates renal dysfunction. Our mouse models recapitulate these phenotypes: significantly increased plasma urea and creatinine levels were measured 4 or more weeks after the development of diabetes (Fig. S1C,D).

Since lower PPi levels cause ectopic calcification in certain genetic disorders and we found lower plasma PPi levels in T1D and T2D patients compared to healthy controls, we also measured plasma PPi levels of diabetic and age‐matched control mice. Our results indicated that circulating PPi significantly decreased in diabetic conditions in all three genetic backgrounds as early as 2 weeks after the development of hyperglycemia (Fig. 1B) and there is an inverse correlation between blood glucose and plasma PPi levels (Fig. 1C). *Abcc6−/−* mice have lower plasma PPi [24], but in diabetic conditions, plasma PPi decreased further (46–57%). In the two wild‐type strains, we investigated (*Abcc6+/+* and CD1), plasma PPi also decreased in diabetic conditions (54–73%), to levels similar to those observed in nondiabetic *Abcc6−/−* mice without significant aorta calcification.

### Calcification of aorta and kidney in diabetic *Abcc6−/−* mice; effect of oral PPi treatment on calcification

To investigate whether diabetes and lower plasma PPi enhanced ectopic calcification in our mouse models, we measured mineralization in the aorta and kidneys. For control purposes, we also measured calcification of the vibrissae capsule in *Abcc6−/−* mice, which is one of the earliest ectopic mineralization symptoms in the PXE model.

**Fig. 2:**
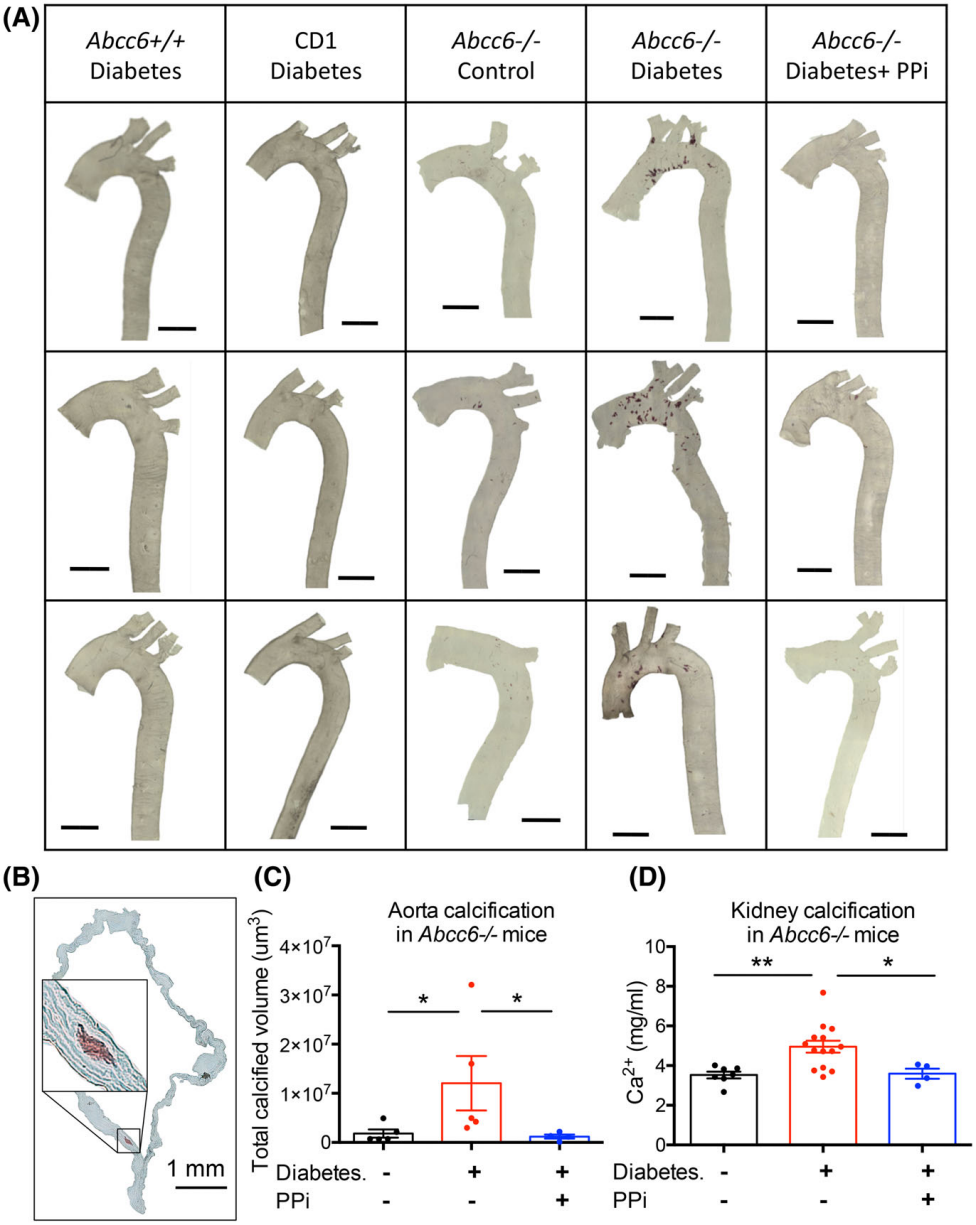
Calcification of aorta and kidney in diabetic *Abcc6−/−* mice and the effect of PPi treatment. (A) Aorta calcification visualized by Alizarin Red staining in diabetic *Abcc6+/+* and CD1 mice and in nondiabetic *Abcc6−/−*, diabetic *Abcc6−/−*, and PPi‐treated diabetic *Abcc6−/−* mice. Scale bar = 2 mm. (B) Cross section of diabetic *Abcc6−/−* aorta, showing calcification in the media layer using Alizarin Red staining. Scale bar = 1 mm. (C) Aorta calcification in control, diabetic, and PPi‐treated diabetic *Abcc6−/−* mice was quantified from 3D microscope images. Large individual differences were detected in diabetic mice (*n* = 4–5). Data were analyzed by two‐tailed Mann–Whitney nonparametric tests; results are expressed as mean ± SEM; *P < 0.05. (D) Kidney calcification measured by colorimetric calcium assay in nondiabetic, diabetic, and PPi‐treated diabetic *Abcc6−/−* mice (*n* = 4–14). Data were analyzed by two‐tailed Mann–Whitney nonparametric tests; results are expressed as mean ± SEM; *P < 0.05, **P < 0.01 (PPi, pyrophosphate).

Whole aortas and cross sections were stained using Alizarin Red S. After imaging (Fig. 2A,B), the extent of calcification was established based on z‐stack confocal microscopy, using a 3D Object Counter plugin developed in the ImageJ program to visualize and analyze microscope data (Fig. 2C). Kidneys and whiskers were minced and incubated in a 0.15 N HCl solution. The calcium content of the supernatant was determined by a colorimetric reaction (Fig. 2D, Fig. S2). STZ‐treated *Abcc6+/+* and CD1 mice show typical signs of diabetes (Fig. S1), but during the investigated period (8 weeks), no calcification was found in the aorta or other soft tissues (Fig. 2A). Diabetic *Abcc6−/−* mice, however, showed mineralization in the medial layer of the aorta and in the kidneys compared to nondiabetic, age‐matched *Abcc6−/−* animals (Fig. 2A–D), but there was no increase in the calcification of the vibrissae capsule (Fig. S2), which is a well‐accepted quantifiable marker of the PXE phenotype [47].

As shown in our previous study [44], administration of PPi via drinking water inhibited the development of chronic calcification in *Abcc6−/−* and *Enpp1−/−* mice, genetic models of PXE and GACI. As the level of PPi was found to be lower in our diabetes animal models, we followed a similar protocol and treated the mice with 0.3 mm PPi for 8 weeks. Our results showed that PPi treatment was sufficient to prevent vascular and kidney calcification in diabetic *Abcc6−/−* mice (Fig. 2A,C,D).

PPi treatment had no effect on serum urea and creatinine levels (Fig. S3).

### Decrease in *Abcc6* and *Enpp1* expression

To elucidate the possible molecular background of reduced plasma PPi in diabetes, the expression levels of *Abcc6* and *Enpp1* genes were measured in the liver as both enzymes control plasma PPi [25]. Our results indicated that there was a modest but significant decrease in hepatic *Abcc6* expression in *Abcc6+/+* and CD1 mice 8 weeks after STZ‐induced hyperglycemia. *Enpp1* expression also decreased in all three investigated groups, but only after 8 or 20 weeks of diabetes (Fig. S4A,B).

### Increased ALP activity, effect of ALP inhibition and insulin treatment

**Fig. 3:**
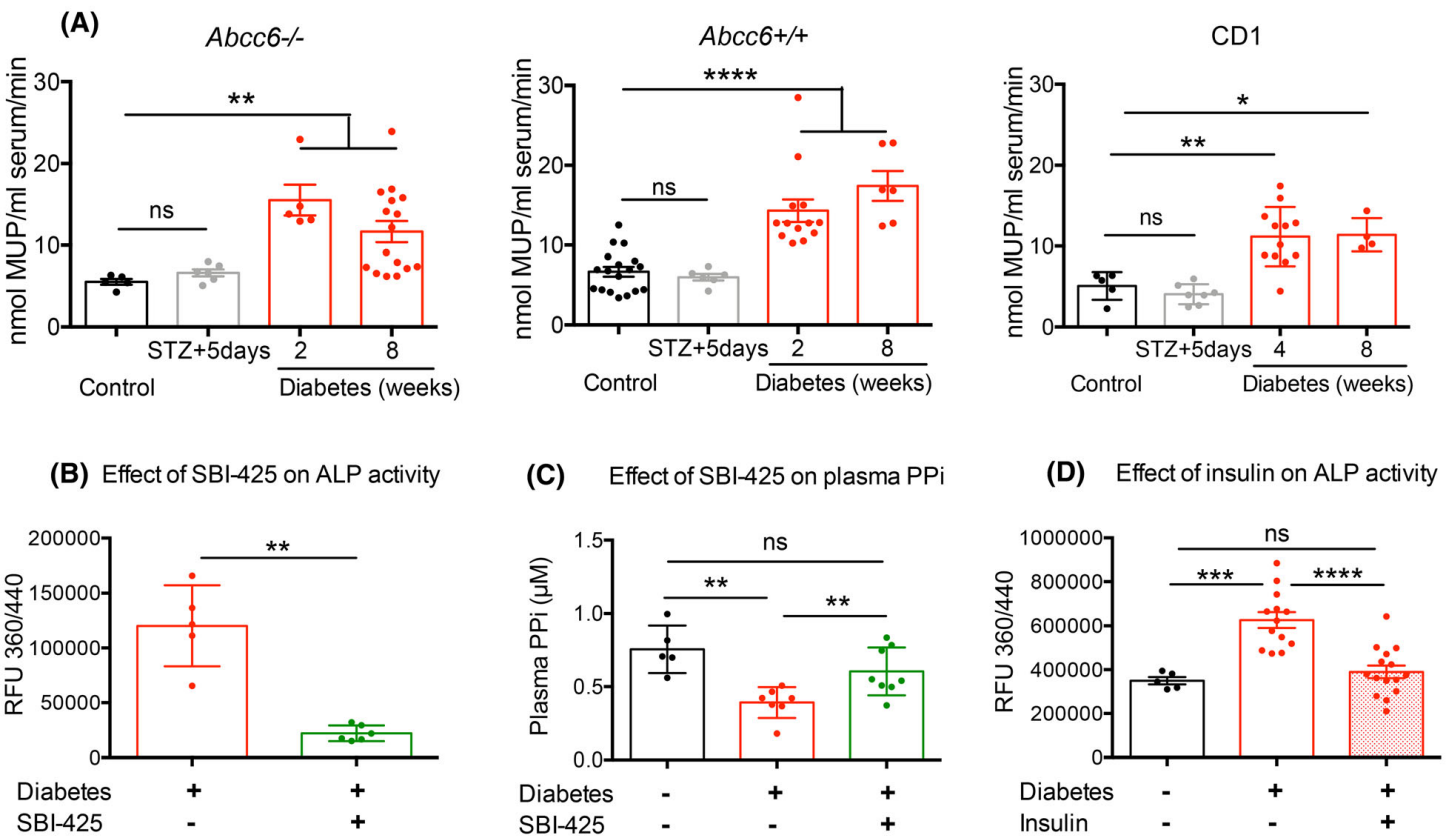
ALP activity and the effect of ALP inhibition and insulin treatment in diabetic mice. (A) ALP activity was measured in the plasma of control and diabetic *Abcc6−/−, Abcc6+/+*, and CD1 mice. ALP activity did not change immediately after STZ injection but increased 2 weeks after diabetes had developed. (B) The effect of SBI‐425, an inhibitor of TNAP, was tested in *Abcc6−/−* mice diabetic for 2 weeks. Mice treated with SBI‐425 have lower plasma ALP activity and (C) higher plasma PPi level. (D) ALP activity did not increase in diabetic *Abcc6+/+* mice when insulin treatment (4 μU·g^−1^ twice a day for 3 weeks) was applied. Data were analyzed by two‐tailed Mann–Whitney nonparametric tests; results are expressed as mean ± SEM; ns, not significant, *P < 0.05; **P < 0.01; ***P < 0.001; and ****P < 0.0001. The scale and unit for ALP activity measurements differ in (A), (B), and (D) because these measurements had to be conducted under different conditions—see in detail in the Methods section (*n* = 4–18) (ALP, alkaline phosphatase; STZ, streptozotocin; TNAP, tissue‐nonspecific alkaline phosphatase; PPi, pyrophosphate).

ALP is another factor that could affect plasma PPi levels. This ecto‐enzyme catalyzes the hydrolysis of PPi into Pi. We found that ALP activity increased as early as 2 weeks after STZ‐induced diabetes and stayed higher compared to control mice throughout the course of the experiment (Fig. 3A). This is in line with our previous finding that PPi levels decreased in the second week of the diabetic condition.

As hepatocytes also express Glut‐2 transporter [48], they are likely susceptible to STZ treatment, which can cause dose‐dependent acute liver damage within a few days and a concomitant increase of ALP activity [49]. As ALP activity was unchanged 5 days post STZ treatment as compared to vehicle‐treated animals, elevated ALP levels were unlikely due to acute injury.

SBI‐425 is a potent orally bioavailable TNAP inhibitor [50]. We designed an *in vivo* experiment to verify that increased ALP activity caused lower plasma PPi: *Abcc6−/−* mice diabetic for 2 weeks were treated with ALP inhibitor SBI‐425, 5 mg·kg^−1^·day^−1^ for 2 days. Plasma ALP activity was decreased in the inhibitor‐treated group compared to vehicle‐treated animals (Fig. 3B), and remarkably, diabetic SBI‐425‐treated *Abcc6−/−* mice showed PPi levels similar to the nondiabetic group (Fig. 3C).

We also investigated the effect of insulin on ALP activity. Diabetic *Abcc6+/+* mice regularly treated with insulin (4 μU·g^−1^ twice a day for 3 weeks) had similar serum ALP activity as age‐matched nondiabetic *Abcc6+/+* animals, while diabetic mice without insulin treatment had elevated ALP activity (Fig. 3D).

## Discussion

In the present work, we found that T1D and T2D patients with cardiovascular manifestations have significantly reduced circulating levels of PPi. Using animal models, we found that circulating PPi and alkaline phosphatase likely play an important role in the diabetes‐related calcification. We demonstrated that the mitigation of soft tissue calcification in diabetic patients is possible with a minimally invasive therapeutic intervention.

To investigate the possible mechanism of diabetes‐related calcification and the role of PPi in this process, we induced diabetes in mice with different genetic backgrounds: *Abcc6−/−* mice susceptible to PXE‐like ectopic calcification and wild‐type control, that is, C57BL/6 (*Abcc6+/+*) and the genetically unrelated wild‐type control CD1. The use of diabetic wild‐type mouse models was essential in order to be able to distinguish whether a molecular change observed in diabetic *Abcc6−/−* mice is due to the lack of Abcc6 expression or due to the diabetic condition. We detected decreased plasma PPi levels in all three strains 2 weeks after hyperglycemia had developed. Plasma PPi levels showed an inverse correlation with blood glucose, confirming the results of Flores‐Roco et al. [38]. This inverse correlation may result from reduced ATP synthesis (the source of PPi) due to mitochondrial dysfunction caused by hyperglycemia [51].

Diabetic *Abcc6−/−* mice showed increased calcification in both aorta and kidney as compared to age‐matched nondiabetic *Abcc6−/−* mice. As there was no change in vibrissae calcification in the experimental animals, the vascular and renal calcification were most likely related to diabetic condition and not the PXE phenotype. The two wild‐type diabetic mouse groups did not develop DM‐related calcification during the investigated period, despite the significant decrease in plasma PPi (>50%). It is important to note that diabetic wild‐type mice had similar plasma PPi levels as nondiabetic *Abcc6−/−* mice, and based on literature data and our observations, this lower level of plasma PPi leads to aortic calcification only at older age (12–18 months) in case of *Abcc6−/−* mice. Therefore, the experimental period (8 weeks) was most likely too short for any significant calcification to develop in the diabetic wild‐type strains [39]. Moreover, it is also well‐documented that a combination of both endogenous and exogenous factors such as the innate immune system, local gene expression, and/or the PPi production/degradation ratio within the connective tissues significantly influence the outcome [52, 53, 54, 55, 56, 57].

We also observed increased ALP activity in all diabetic mice 2 weeks post hyperglycemia onset. As inhibiting ALP restored baseline PPi levels in diabetic mice, it seems probable that increased ALP activity contributed to lowering PPi levels and enhanced ectopic calcification susceptibility in the context of diabetes. Remarkably, elevated serum ALP levels in a diabetic rat model could be normalized with insulin replacement [58]. Our results also showed that regular insulin treatment in diabetic mice was also aimed to maintain ALP activity at normal levels. In the plasma of T1D and T2D patients, we did not measure an elevation of ALP activity, most likely because the majority of the recruited patients were under diabetes medication (insulin, metformin, or sulphonylureas).

Overall, our results are consistent with the recent findings by Flores‐Roco *et al*. [38], showing that high blood glucose level indeed led to increased ALP activity and decreased PPi level. With our experimental process, we showed that hyperglycemia leads to enhanced ectopic calcification *in vivo*, and we were able to determine the sequence of DM‐related molecular changes happening. Diabetes first led to an increase in ALP activity, which lowered plasma PPi level. Changes in renal function and expression levels of PPi metabolism‐related genes occurred at later stages.

Treating diabetic *Abcc6−/−* mice with 0.3 mM PPi in the drinking water prevented the development of vascular and renal calcification. This result is quite remarkable as the bioavailability of oral PPi is very low [59]. More importantly, the reduced levels of circulating PPi in diabetic patients and mice and the effective compensation with oral delivery strongly suggested a determining role for PPi in calcification associated with DM and also indicated that PPi treatment could be an effective preventive therapy for diabetes‐related vascular calcification.

With that in mind, our previous preclinical studies [44, 60] showed that PPi supplementation is an effective approach to prevent ectopic calcification, which has promoted a clinical trial in France (NCT04868578: PPi Supplementation for Fight Ectopic Calcification in PXE) with special attention to arterial calcification [61]. The clinical observations of this trial on the efficacy and tolerability of oral PPi could well be a stepping stone for a later use of PPi for diabetic patients. Three other trials are registered with the aim of indirectly increasing plasma PPi concentration [NCT05569252, NCT04660461, NCT05030831] in PXE [62]. However, it is important to note that PPi treatment is unable to remove the already existing hydroxyapatite precipitate, and it is likely that other molecular mechanisms are influencing the development of ectopic calcification in diabetic patients.

In summary, we found both in humans and mice that enhanced vascular calcification in DM is at least in part related to a deficit in circulating PPi resulting from elevated ALP activity in the circulation. Deterioration of kidney function and the decreased expression of *Abcc6* or *Enpp1* are not the likely causes of decreased plasma PPi, as these changes occur at later stages of diabetes in mice. The preclinical data presented here showed that a simple supplementation of PPi represents a potentially effective therapeutic solution.

## Conflict of interest

AV is co‐inventor on patent applications related to the use of pyrophosphates for therapeutic applications. Application entitled “Oral Pyrophosphate For Use In Reducing Tissue Calcification” US 16/333856 and EP17781568.5. Other authors have no conflict of interest.

## Author contributions

Conceptualization: VP, KF, AV, OLS, JB, MH. Methodology: KF, VP, EK, NT, AKe. Investigation: KF, VP, EK, LK, ZG, AKu. Visualization: NT, AKe, EK. Chemical synthesis: ZR. Human data collection: MH, ÁD. Supervision: VP, AV. Writing—original draft: VP, KF. Writing—review and editing: VP, KF, EK, AV, OLS. VP is the guarantor of this work and, as such, has full access to all of the data in the study and takes responsibility for the integrity of the data and the accuracy of the data analysis.

## Supporting information


**Fig. S1.** Changes in blood glucose, urine volume and level of plasma urea and creatinine in diabetic mice.
**Fig. S2.** Calcification of vibrissae capsules in *Abcc6−/−* mice.
**Fig. S3.** Plasma urea and creatinine levels in PPi‐treated mice.
**Fig. S4.** Expression levels of hepatic *Abcc6* and *Enpp1*, key modulators of plasma PPi level.
**Table S1.** Clinical data of human diabetic patients.

## Data Availability

All data supporting the findings of this study are available within the paper and its Supplementary Information.
