# Supplementary material for: Diabetes‐induced vascular calcification is associated with low pyrophosphate and its oral supplementation prevents calcification in diabetic mice
Source: FEBS Open Bio. 2025 Oct 13;16(3):474–86. doi: 10.1002/2211-5463.70141 (PMC12955752; doi:10.1002/2211-5463.70141)
Supplement: Supplementary file 1 — Fig. S1. Changes in blood glucose, urine volume and level of plasma urea and creatinine in diabetic mice. Fig. S2. Calcification of vibrissae capsules in Abcc6−/− mice. Fig. S3. Plasma urea and creatinine levels in PPi‐treated mice. Fig. S4. Expression levels of hepatic Abcc6 and Enpp1, key modulators of plasma PPi level. Table S1. Clinical data of human diabetic patients. [file FEB4-16-474-s001.pdf]

Supplementary table and figures with legends:

	age (years)	T1D	T2D	**CAD**	**AMI**	**stroke**	**CAAD**	**PAD**	**HT**	ALP (U/L)
DP1	57	0	1	1	0	0	0	0	1	NA
DP2	72	0	1	1	1	0	0	1	1	NA
DP3	64	0	1	1	0	0	1	0	1	108
DP4	63	0	1	1	1	0	0	0	1	99
DP5	77	0	1	1	1	0	1	0	1	36
DP6	63	0	1	1	1	0	0	1	1	NA
DP7	64	0	1	1	1	0	0	0	1	57
DP8	78	0	1	0	0	0	1	0	0	91
DP9	67	1	0	1	1	0	0	0	1	77
DP10	54	0	1	0	0	0	0	1	1	NA
DP11	67	0	1	1	0	0	0	0	1	NA
DP12	73	0	1	1	1	1	0	0	1	55
DP13	65	0	1	1	1	0	0	1	1	78
DP14	71	0	1	1	0	0	0	0	1	75
DP16	71	0	1	1	1	0	1	1	1	66
DP17	73	0	1	1	0	0	0	0	1	NA
DP18	71	1	0	0	0	0	1	0	1	175
DP19	63	0	1	0	0	0	1	1	0	57
D20	55	0	1	1	0	0	0	0	1	73
D21	86	0	1	0	0	0	0	1	1	89
D22	63	0	1	1	1	0	1	0	1	57
D23	79	0	1	1	0	0	0	0	1	49
D24	77	0	1	1	0	0	0	0	1	96
D25	83	0	1	1	0	0	0	0	1	82
D26	76	0	1	1	0	1	1	0	1	114
D27	67	0	1	0	0	0	1	1	1	55
D28	61	0	1	1	1	0	0	0	1	59
D29	73	1	0	0	0	0	0	1	1	NA
D30	70	1	0	1	1	0	1	1	1	127
D31	56	0	1	0	0	1	1	0	1	NA
D32	75	0	1	NA	NA	NA	NA	NA	NA	NA
D33	74	0	1	1	0	0	0	1	1	178
D34	77	0	1	1	0	1	1	0	1	123
D35	86	1	0	1	1	0	0	0	1	57
D36	73	0	1	1	0	1	0	0	1	51
D37	78	0	1	0	0	0	0	1	1	120
D38	82	0	1	1	1	0	0	0	0	NA
D40	40	1	0	1	0	1	0	0	1	NA
D41	71	0	1	0	0	0	1	1	1	83
D42	67	0	1	1	0	0	0	0	1	96
D43	63	0	1	0	0	0	1	0	1	NA
D44	70	0	1	1	0	0	1	1	1	NA
D45	66	1	0	0	0	1	0	1	1	86
D46	80	0	1	1	0	0	1	0	1	82
D47	72	0	1	1	0	0	1	0	1	NA
D48	53	1	0	0	0	0	0	0	1	79
D49	81	0	1	1	0	0	0	1	1	66
D50	54	1	0	0	0	1	0	0	1	NA
D51	60	1	0	0	0	0	1	1	1	NA
D52	56	0	1	0	0	0	0	1	1	NA
D53	80	0	1	0	0	0	1	0	1	NA
D54	76	0	1	1	0	0	0	0	1	NA
D55	77	0	1	1	0	0	1	0	1	76
D56	56	1	0	0	1	0	0	0	1	72
D57	65	0	1	1	1	0	0	0	1	67
D58	70	0	1	1	0	0	0	1	1	NA
D59	68	0	1	0	1	0	0	0	1	44
D60	67	0	1	1	1	0	1	1	1	60
D62	74	0	1	0	0	0	0	0	1	NA
D63	55	1	0	0	1	0	0	0	1	NA
D64	60	0	1	0	0	0	1	1	1	NA
				38	19	8	22	21	57	

Supplementary table 1: **Clinical data of human diabetic patients.** Available data (age, type of diabetes, the presence of other cardiovascular complications and plasma ALP activity) from the diabetic cohort is summarized in the table. CAD: coronary artery disease; AMI: acute myocardial infarction; CAS: carotid artery stenosis; PAD: peripheral arterial disease; HT: hypertension; ALP: plasma alkaline phosphatase activity.

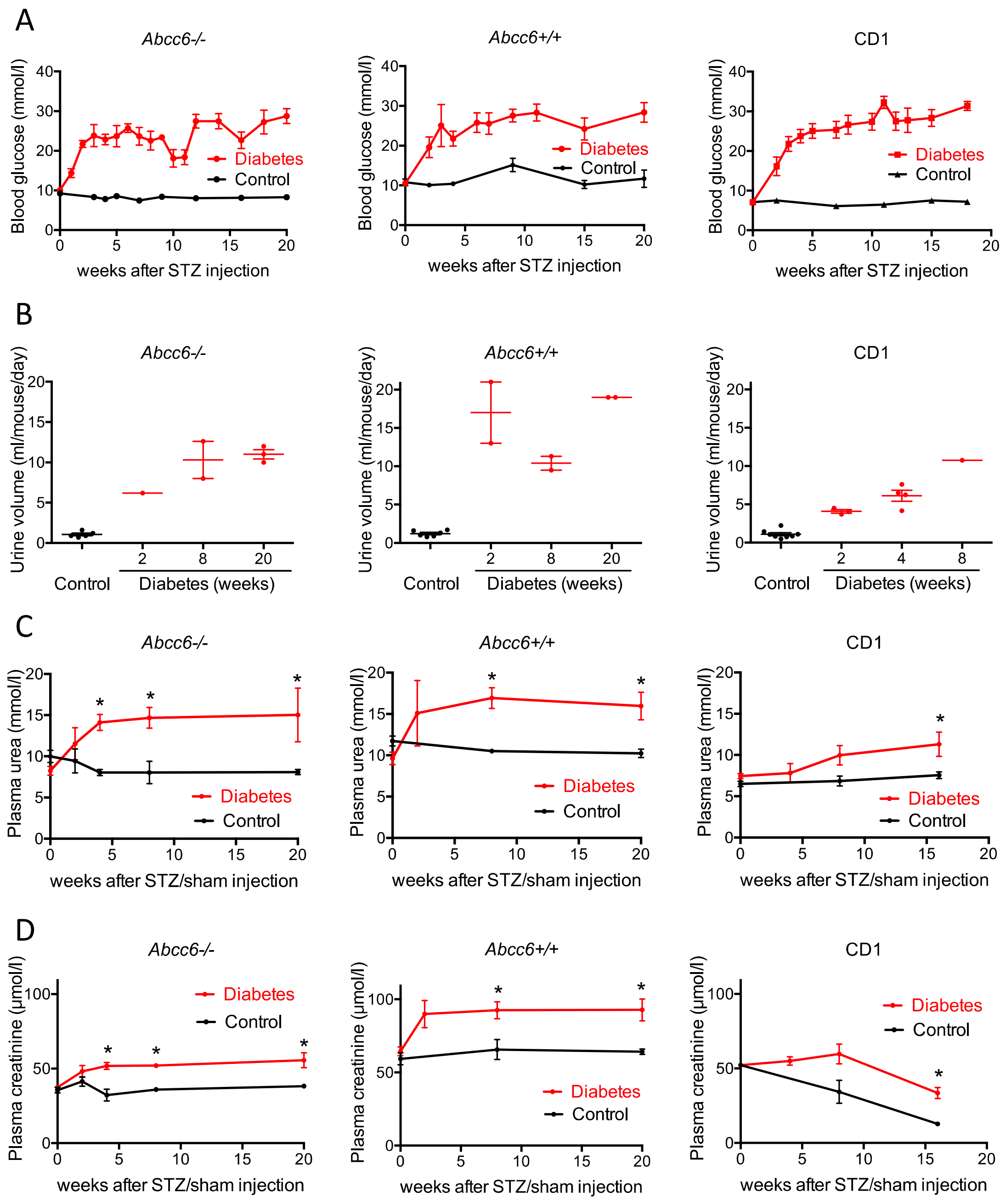

Supplementary figure 1: **Changes in blood glucose, urine volume and level of plasma urea and creatinine in diabetic mice.** (A) Streptozotocin (STZ) injection induced increased blood glucose level in *Abcc6-/-, Abcc6+/+* and in CD1 mice, as expected under diabetic conditions (n=5). (B) Volume of urine significantly increased in diabetic condition in all 3 investigated strains, even after 2 weeks of diabetic condition (n=1-8). Plasma levels of (C) urea and (D) creatinine also increased during diabetes – indicating renal failure – but only after 4 weeks or later. Data were analyzed by two-tailed Mann–Whitney nonparametric tests, results are expressed as mean ± SEM; *p < 0.05. (n=3-9)

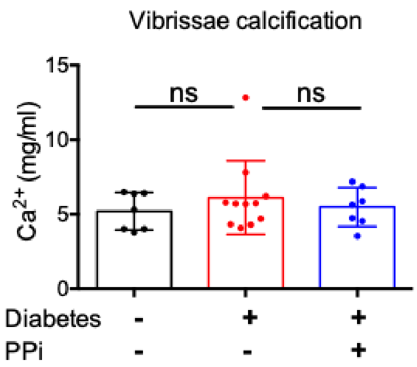

Supplementary figure 2: **Calcification of vibrissae capsules in *Abcc6-/-* mice.** Vibrissae calcification was determined in non-diabetic *Abcc6-/-* mice and in diabetic (8 weeks) *Abcc6-/-* mice with or without PPi treatment. Calcium content was measured by colorimetric calcium assay. Data were analyzed by two-tailed Mann–Whitney nonparametric tests, results are expressed as mean ± SEM; ns = not significant (n=7-14) (PPi: pyrophosphate)

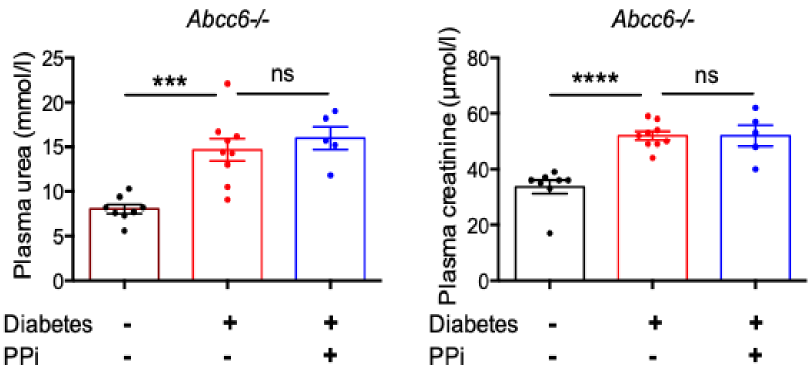

Supplementary figure 3: **Plasma urea and creatinine levels in PPi-treated mice**. Plasma urea and creatinine levels, indicators of renal function, did not change due to 8 weeks of PPi treatment in *Abcc6-/-* diabetic mice compared to *Abcc6-/-* diabetic mice receiving no PPi supplementation. Data were analyzed by two-tailed Mann–Whitney nonparametric tests, results are expressed as mean ± SEM; ns = not significant; ***$p < 0.001$; ****$p < 0.0001$. (n=5-9) (PPi: pyrophosphate)

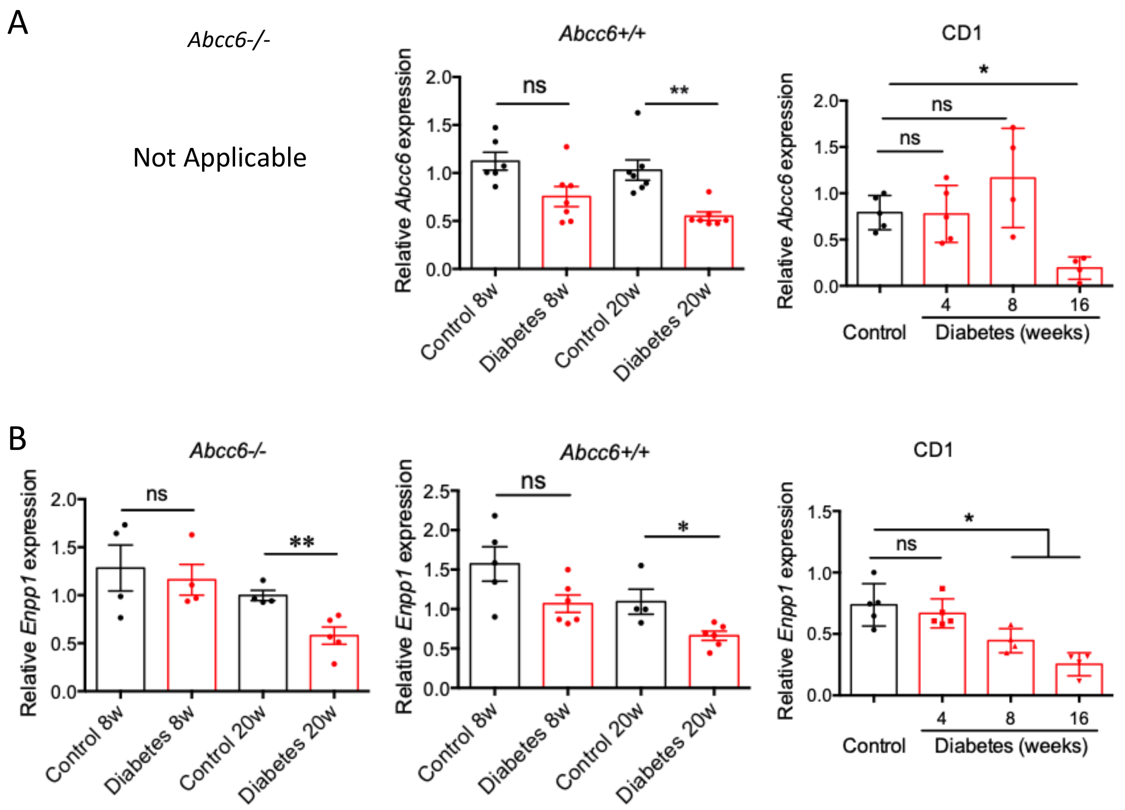

Supplementary figure 4: **Expression levels of hepatic *Abcc6 and Enpp1*, key modulators of plasma PPi level.** Expression levels of (A) *Abcc6* and (B) *Enpp1* were measured in the liver of non-diabetic and diabetic mice. *Abcc6* expression was measured in *Abcc6+/+* and CD1 mice, while *Enpp1* expression was measured in all 3 strains. Both genes were decreased in diabetic condition, but only after 8, 16 or 20 weeks of diabetes. Data were calculated using the delta-delta threshold method and analyzed by Student's t-test. Differences between relative gene expressions greater than 1.5-fold were considered statistically significant. ns = not significant; *$p < 0.001$; **$p < 0.0001$. (n=4-7). (PPi: pyrophosphate; w: weeks)
